# Lung inflammation changes and oxidative stress induced by cigarette smoke exposure in guinea pigs affected by *Zataria multiflora* and its constituent, carvacrol

**DOI:** 10.1186/s12906-015-0574-y

**Published:** 2015-03-03

**Authors:** Mohammad Hossein Boskabady, Leila Gholami Mahtaj

**Affiliations:** Neurogenic Inflammation Research Centre and Department of Physiology, School of Medicine, Mashhad University of Medical Sciences, Mashhad, Iran; Pharmaceutical Research Centre and Department of Physiology, School of Medicine, Mashhad University of Medical Sciences, Mashhad, Iran

**Keywords:** *Zataria multiflora* Boiss L, Carvacrol, COPD, Thiol groups, Oxidative stress, Lung inflammation

## Abstract

**Background:**

Chronic obstructive pulmonary disease (COPD) is an epidemic and progressive health problem which is mainly a consequence of cigarette smoking, and associated with lung inflammation. Anti-inflammatory property of *Zataria multiflora* (*Z. multiflora*) and its constituent, carvacrol was shown in various inflammatory disorders previously. Therefore, in the present study, the effects of the plant and its constituent, carvacrol, on lung inflammation changes and oxidative stress, in guinea pigs model of COPD were evaluated.

**Methods:**

Nine groups of animals including control, COPD, COPD + drinking water containing three concentrations of extract of *Z. multiflora* (0.4, 0.8, and 1.6 mg/mL), COPD + drinking water containing three concentrations of carvacrol (60, 120, and 240 μg/mL), and COPD + dexamethasone (50 μg/mL) were studied. For inducing COPD, animals were exposed to cigarette smoke for 3 months. Thiol groups, IL-8, total and differential WBC were measured in broncho-alveolar lavage fluid (BALF) (n = 6 for each group).

**Results:**

Total WBC, eosinophils, and neutrophils counts as well as the levels of IL-8 in BALF were significantly increased but thiol group was decreased in COPD compared to the control group (p < 0.05 to p < 0.001). Total WBC and IL-8 in all treated COPD groups, thiol group, eosinophils and neutrophils counts in treated groups with dexamethasone and two higher concentrations of the *Z. multiflora* and carvacrol were significantly improved compared to non-treated COPD group (p < 0.05 to p < 0.001). Lymphocyte count in treated groups with dexamethasone, highest concentration of *Z. multiflora*, and two higher concentration of carvacrol was also significantly higher than non-treated group (p < 0.05 to p < 0.001).

**Conclusions:**

A preventive effect of *Z. multiflora* extract and its constituent carvacrol on lung inflammation changes and oxidative stress in animal model of COPD was suggested.

## Background

Chronic obstructive pulmonary disease (COPD), with smoking as an important risk factor for its development [1] is an epidemic and progressive world health problem [2].

Inflammatory processes, oxidative damage, proteolysis, vascular remodeling, and lung tissue changes of COPD are well-documented changes in this disease [3]. CD68+ monocytes or macrophages in the bronchial mucosa [4], TNFα, interleukin-8, and macrophage inflammatory protein-1α (MIP-1α) which produce a positive inflammatory chain are increased in COPD [5]. An increase in the number of neutrophils is also observed in severe COPD patients [6]. It was shown that antioxidants may have a role in the treatment of COPD patients [7]. Animal model of COPD using cigarette smoke exposure has been induced in guinea pigs which leads to emphysematous lung destruction and lung inflammation [8-10].

*Z. multiflora* is a perennial plant [11] wich mainly grows in Iran, Pakistan, and Afghanistan [12]. *Z. multiflora* is used in Iranian traditional medicine for antiseptic, analgesic, and carminative properties [13]. Different pharmacological effects including analgesic [14,15], antioxidant [16], antitussive [17], and anti-inflammatory effects [15] were shown for this plant. In our previous studies, the inhibitory effect of the extracts from *Z. Multiflora* and carvacrol on histaminergic (H_1_) [18] and muscarinic receptors [19-21] and its stimulatory effect on β-adrenoceptors [22] as well as the effect of the plant on systemic inflammation of cigarette smoke exposed animals [23] were demonstrated. The medicinal use of all plants containing carvacrol including *Z. multiflora* on respiratory diseases were also described [24,25]. In addition, various pharmacological effects including anti-inflammatory [26,27] and relaxant properties [28,29] were shown for carvacrol.

Therefore, in the present study, the effects of *Z. multiflora* and carvacrol on lung inflammatory changes and oxidative stress on guinea pig model of COPD have been examined.

## Methods

### Plant and extract

*Z. multiflora* was collected from mountains in the mine fluorine area, in the region between Tabas and Yazd, centre of Iran, and identified by Mr. Joharchi and a control sample was kept in the Herbarium of the Faculty of Sciences, Ferdowsi University of Mashhad (herbarium number: 35314). Hydro-ethanolic extract of 125 g dried shoots and powdered *Z. multiflora* was prepared by mixing it with 875 mL of 50% ethanol and shaking for 72 hours at room temperature. The extract was then passed through the filter paper and the solvent was removed under reduced pressure. The yield extract from the plant was 18.7 g. The dried extract was collected and kept at refrigerator temperature for experimental purpose.

### Characterization of the extract of *Zataria multiflora*

**Figure 1:**
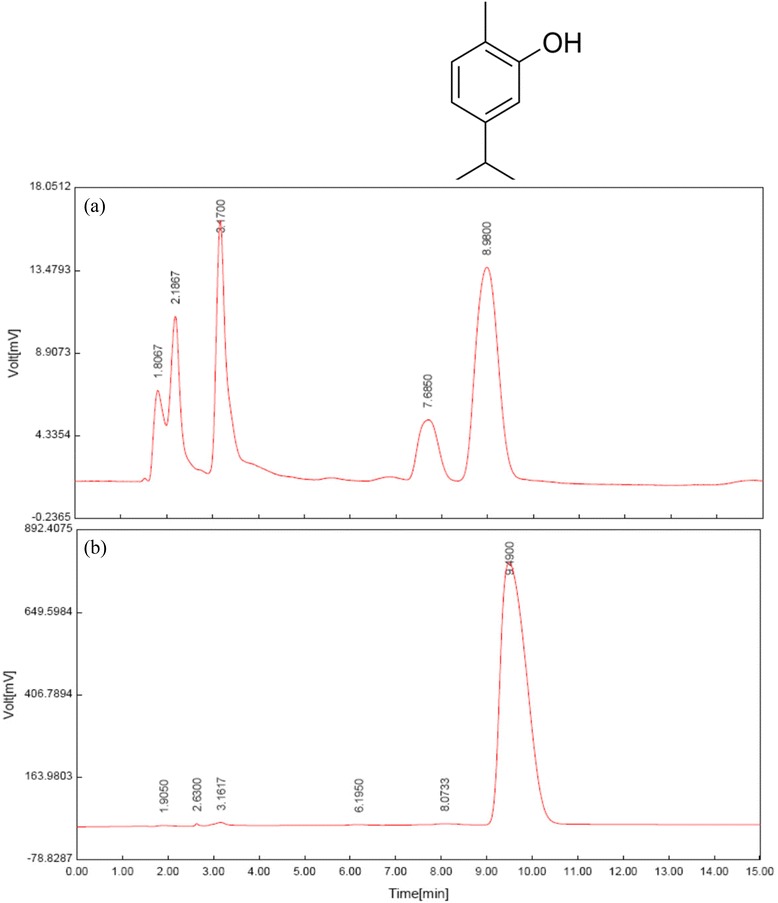
**Fingerprint of (a) the extract of** ***Z. multiflora*** **(50 μg/mL) and (b) chromatographic profile of pure carvacrol (C**_**10**_ **H**_**14**_ **O, 5/1000) with retention time of about 9 min.** MW = 150.217 (Ref [22]).

The characteristic of the extract of this plant was identified using HPLC (Waters 474, Waters Corporation, MA, USA) fingerprint as described in our previous study [22], (Figure 1).

### Animals and groups

Fifty four guinea pigs of both sexes (600–800 g) were purchased from Razi Institute, Mashhad, Iran and were kept at 22 ± 2°C on a 12 h light/dark cycle during the study period. Experiments were performed in compliance with the regulations of the Institute of Laboratory Animals Resources, Commission on Life Sciences [30]. Animals were divided into nine groups in a random order which received drinking water as follow (for each group, n = 6):

1. Control group: the animals were exposed to ambient air and received drinking water alone.
2. COPD group: the animals were exposed to cigarette smoke and received drinking water alone.
3. COPD + dexamethasone (Sigma Chemical Company, Ltd, Poole, Dorset BH12 4QH, UK, purities ≥97%): the animals were exposed to cigarette smoke and received drinking water containing 50 μg/mL dexamethasone.
4. COPD + *Z. multiflora* dose 1: the animals were exposed to cigarette smoke and received drinking water containing 0.4 mg/mL of the extract.
5. COPD + *Z. multiflora* dose 2: the animals were exposed to cigarette smoke and received drinking water containing 0.8 mg/mL of the extract.
6. COPD + *Z. multiflora* dose 3: the animals were exposed to cigarette smoke and received drinking water containing 1.6 mg/mL of the extract.
7. COPD + carvacrol (Sigma Chemical Company, Ltd, Poole, Dorset BH12 4QH, UK, purities ≥98%) dose 1: the animals were exposed to cigarette smoke and received drinking water containing 60 μg/mL carvacrol.
8. COPD + carvacrol dose 2: the animals were exposed to cigarette smoke and received drinking water containing 120 μg/mL carvacrol.
9. COPD + carvacrol dose 3: the animals were exposed to cigarette smoke and received drinking water containing 240 μg/mL carvacrol.

The extract, carvacrol, or dexamethasone, were added to the drinking water daily which was freely available to animals during the period of cigarette smoke exposure (3 months). The volume of uptake of drinking water was checked regularly which was about 100 mL/day for each guinea pig in all of the groups. Although carvacrol and ethanolic extract are lipophilic compounds but due to their low concentration used in the present study, they were well emulsified in drinking water.

### Exposure of animals to cigarette smoke

Guinea pigs were exposed to cigarette smoke in order to induce animal model COPD, as previously described [31,32]. Briefly, animals were placed in two chamber box; a smaller closed chamber for the head with a hole at its top for delivering of cigarette smoke to the animal’s head and a larger opened part for the body of guinea pigs. The animal’s neck was fixed between the two chambers by a sliding pyrepex sheet. Each animal was initially exposed to one cigarette per day which was gradually increased to a maximum of 5 cigarettes per day over a period of 20 days. The animals were exposed to smoke of 5 cigarette/day, 5 days per week (Magna: Nicotine = 5, tar = 6, the cigarettes’ filters weren’t removed) for 70 days. Therefore, each animal was exposed to cigarette smoke for a period of 3 consecutive months. Cigarette smoke was drawn into a 20 mL syringe and exhausted at a rate of two puffs per minute into the animals’ head chamber. The exposure of animals to the smoke of each cigarette was taken 8–9 minutes and there was a 10-minute break between the two cigarettes. The study was approved by the Ethical Committee of Mashhad University of Medical Sciences (August 2012, approval No. 900799).

### Biochemical assay

Broncho-alveolar lavage fluid (BALF) was prepared from the lung following animal sacrificing and opening of their chest. A cannula was placed into the trachea and lungs were lavaged with 2 mL of saline for 5 times (total 10 mL). BALF was then centrifuged at 2500 × g for 10 min and the supernatant was collected for measurement of thiol group and IL-8 concentration.

Total thiol group was measured as previously described [33]. In summary, 1 mL Tris-EDTA buffer was added to 50 μL of BALF and sample absorbance was read at 412 nm against Tris-EDTA buffer alone (A1). Then, 20 μL 2,2-dinitro-5,5′-dithiodibenzoic acid (10 mM in methanol) reagent (DTNB) was added to the mixture. DTNB reacts with the SH groups to produce a yellow colored complex with peak absorbance at 412 nm. The sample absorbance was read again after 15 min (A2). Total thiol concentration was calculated according to the following formula:

$$ \mathrm{Total}\ \mathrm{thiol}\ \left(\mathrm{m}\mathrm{M}\right) = \left(\mathrm{A}2\hbox{-} \mathrm{A}1\hbox{-} \mathrm{blank}\right) \times 1.07/0.05 \times 13.6. $$

The level of IL-8 was measured using a double antibody sandwich enzyme-linked immunosorbent assay (ELISA) kit (Hangzhou Eastbiopharm Co., Ltd., Hangzhou, China) according to the manufacturer’s protocol.

### Total and differential WBC measurement

BALF sample was taken from the lung and then transferred into the test tube containing anticoagulant EDTA. Total WBC was counted in duplicate in a hemocytometer (in a Burker chamber) in blood stained with Turk solution. Differential cell counts were performed on thin slide, prepared with smearing BALF sample using Wright-Giemsa’s stain. According to staining and morphological criteria, differential cell analysis was carried out under a light microscope by counting 100 cells and calculating the percentage of each cell type.

### Statistical analysis

Data were expressed as mean ± SEM. Comparisons between the results of COPD and control groups were performed using unpaired t-test. The data of treated groups were also compared with non-treated COPD group using unpaired t-test. The comparisons between the data of animals treated with three concentrations of *Z. multiflora* and carvacrol were performed using ANOVA with Tukey-Kramer post test. Significance was accepted at p < 0.05. Statistical analyses were made using GraphPad Instat version 3.00 (GraphPad Software, San Diego, California, USA).

## Results

**Figure 2:**
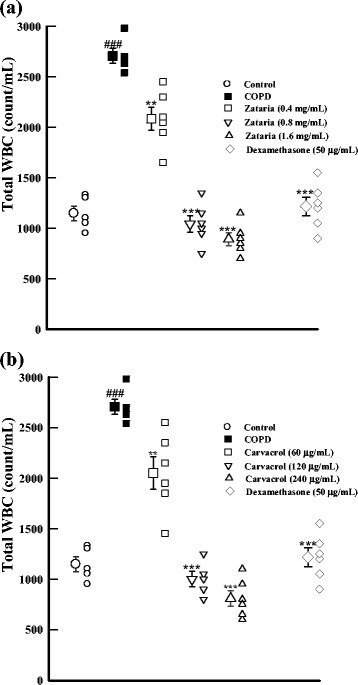
**Total WBC count (in 1 mL) in BALF of control, COPD, and COPD treated with dexamethasone, three concentrations of** ***Z. multiflora*** **(a), and carvacrol (b).** ###; p < 0.001 compared to control group. **; p < 0.01, ***; p < 0.001 compared to non-treated COPD group.

**Figure 3:**
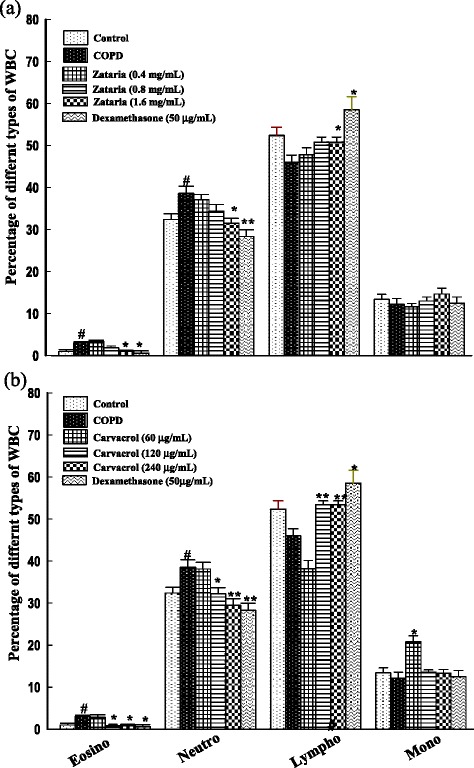
**Differential WBC counts as the percentage of total WBC in BALF of control, COPD, and COPD treated with dexamethasone, three concentrations of** ***Z. multiflora*** **(a), and carvacrol (b).** #; p < 0.05 compared to control group. *; p < 0.05, **; p < 0.01 compared to non-treated COPD group.

Total WBC (p < 0.001), neutrophils and eosinophils counts (p < 0.05 for both cases) in BALF of COPD were significantly higher compared to control group (Figures 2 and 3). Total WBC number in all treated groups and eosinophil and neutrophil percentage in the treated groups with dexamethasone and highest concentration of the extract and two higher concentrations of carvacrol were significantly improved compared to COPD group (p < 0.05 to p < 0.001). In addition, lymphocyte percentage in treated groups with dexamethasone, highest concentration of *Z. multiflora* and two higher concentrations of carvacrol were significantly increased compared to COPD group (p < 0.05 to p < 0.01, Figures 2 and 3).

**Figure 4:**
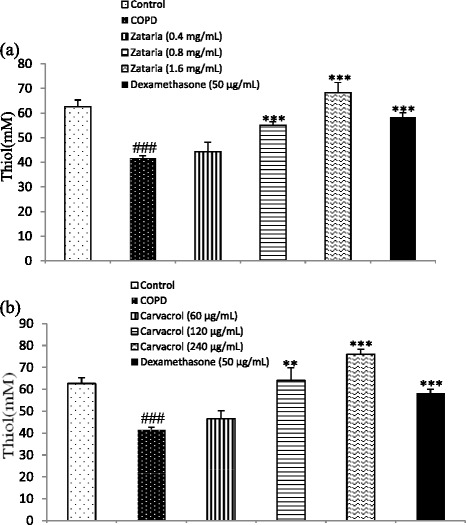
**The level of thiol group in BALF of control, COPD, and COPD treated with dexamethasone, three concentrations of** ***Z. multiflora*** **(a), and three concentrations of carvacrol (b).** ###; p < 0.001 compared to control and **; p < 0.01, ***; p < 0.001 compared to non-treated COPD group.

The concentration of thiol group in BALF of COPD was significantly decreased compared to control group (p *<* 0.001). In COPD groups treated with dexamethasone, two higher concentrations of *Z. multiflora* and carvacrol, the value of thiol group was significantly increased compared to the COPD animals (p *<* 0.01 to p < 0.001, Figure 4).

**Figure 5:**
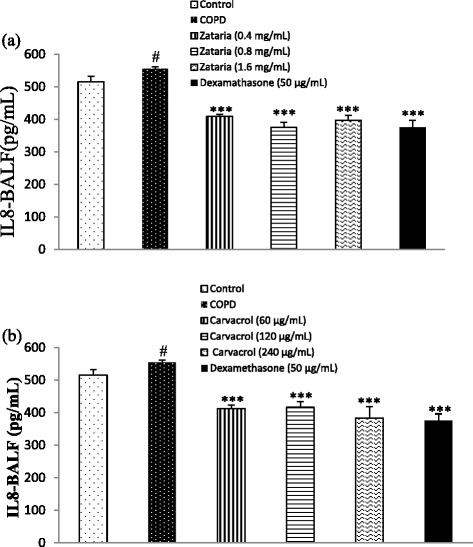
**The level of IL-8 in BALF of control, COPD, and COPD treated with dexamethasone, three concentrations of** ***Z. multiflora*** **(a) and carvacrol (b).** #; p < 0.05 compared to control group. ***; p < 0.001 compared to non-treated COPD group.

The level of IL-8 in BALF of COPD group was significantly increased compared to control group (p < 0.05). In treated groups with dexamethasone, three concentrations of *Z. multiflora* and carvacrol, the level of IL-8 was significantly lower than COPD group (p < 0.001, for all cases, Figure 5).

Although the effects of treatment with low concentration of the extract and carvacrol on total WBC, eosinophil, neutrophil, and thiol group were significantly lower than the effect of their highest concentration, on thiol group, it was higher compared to the effect of dexamethasone (p < 0.05 to p < 0.001, Tables 1 and 2). However, there was no significant difference in the level of IL-8 in BALF between treated groups with different concentrations of the extract and carvacrol compared to dexamethasone (Table 2). In addition, the effects of medium and high concentrations of the extract on total and differential WBC counts were not significantly different than the effect of dexamethasone (Table 1).

**Table 1:** **Values of total (number/mL) and differential (%) WBC in BALF of COPD treated with** ***Z. multiflora*** **, carvacrol, and dexamethasone (Dexa.)**

**Parameter**	**Zataria (0.4 mg/mL)**	**Zataria (0.8 mg/mL)**	**Zataria (1.6 mg/mL)**	**Carvacrol (60 μg/mL)**	**Carvacrol (120 μg/mL)**	**Carvacrol (240 μg/mL)**	**Dexa. (50 μg/mL)**
**Total WBC**	2.08 ± 0.11+++	1.04 ± 0.82###	0.89 ± 0.62###	2.05 ± 0.15+++	1.00 ± 0.75###	0.80 ± 0.76###	1.21 ± 0.92
**Eosinophil**	3.33 ± 0.33++	1.83 ± 0.40+,#	1.00 ± 0.25##	2.83 ± 0.60++	0.80 ± 0.37#	0.83 ± 0.307###	0.83 ± 0.40
**Neutrophil**	37.16 ± 1.19+	34.33 ± 1.64	31.50 ± 1.17#	38.16 ± 1.53++	32.20 ± 1.46#	29.50 ± 1.58##	30.83 ± 1.01
**Monocyte**	11.66 ± 0.76	130 ± 0.96	14.66 ± 1.40	12.66 ± 1.54	13.60 ± 0.51	13.33 ± 0.91	14.50 ± 1.25
**Lymphocyte**	47.83 ± 1.64	50.83 ± 1.16	52.83 ± 1.01#	46.33 ± 1.85	53.4 ± 0.92#	56.33 ± 1.28###	53.83 ± 1.30

Data are presented as mean ± SEM. WBC is expressed as its count in 1 mL BALF and those of each type of WBC is the percentage of total WBC. +; p < 0.05, ++; p < 0.01, +++; p < 0.001 compared to the effect of dexamethasone using unpaired t-test. #; p < 0.05, ##; p < 0.01. ###; p < 0.001, compared to the effect of low concentration of *Z. multiflora* and carvacrol, using (ANOVA) with Tukey–Kramer multiple post test.

**Table 2:** **The levels of thiol groups (mM) and IL-8 (pg/mL) in BALF of COPD animals treated with** ***Z. multiflora*** **, carvacrol, and dexamethasone (Dexa.)**

**Parameter**	**Zataria (0.4 mg/mL)**	**Zataria (0.8 mg/mL)**	**Zataria (1.6 mg/mL)**	**Carvacrol (60 μg/mL)**	**Carvacrol (120 μg/mL)**	**Carvacrol (240 μg/mL)**	**Dexa. (50 μg/mL)**
**Thiol (mM)**	44.33 ± 1.84+++	55.15 ± 1.27###	68.34 ± 4.06+,###,¶	46.66 ± 3.52+¶	64.08 ± 5.81#	76.01 ± 2.32+++,###	58.20 ± 1.82
**IL-8 (pg/mL)**	409.15 ± 6.84	375.21 ± 15.31	396.74 ± 15.84	412.53 ± 11.16	416.66 ± 16.91	383.46 ± 34.35	375.02 ± 21.15

Data are presented as mean ± SEM. +; p < 0.05, +++; p < 0.001, compared to the effect of dexamethasone using unpaired t-test. #; p < 0.05, ###; p < 0.001, compared to the effect of low concentration of *Z. multiflora* and carvacrol, and ¶; p < 0.05, compared to the effect their medium concentration using (ANOVA) with Tukey–Kramer multiple post test.

There was no significant difference between the effect of treatment using different concentrations of carvacrol with their corresponding extract concentration (Tables 1 and 2).

The effects of treated COPD groups with highest concentration of the extract and carvacrol on all measured parameter except for IL-8 was significantly higher than their lowest concentration (p < 0.05 to p < 0.001, Tables 1 and 2). The effects of medium concentration carvacrol on all parameters except for IL-8 and the effect of medium extract concentration on total WBC and eosinophil counts and thiol group was also significantly higher than the effect of their lowest concentration (p < 0.05 to p < 0.001, Tables 1 and 2). In addition, the effect of highest extract concentration on thiol group was also significantly higher than its medium concentration (p < 0.05, Table 2).

## Discussion

The exposure of animals to cigarette smoke resulted in a significant increase of BALF total WBC, eosinophils and neutrophils counts, and IL-8 but decrease in thiol group compared to non-exposed control animals. Several previous studies have shown the induction of animal model of COPD by cigarette smoke exposure using similar method used in the present study [10,31,32]. It was also shown that inflammatory cells play an important role in COPD including macrophages and neutrophils [34]. Eosinophils are a source of cytokines IL-3, 4, 5, 6, 8, and eosinophil-derived neurotoxin as well as eosinophil peroxidase that have a role in the pathogenesis of COPD. An increase in IL-8 level in smokers with COPD compared to healthy people was also observed previously [34].

According to the results of the present study, oxidative stress was increased in the COPD group in their BALF measured by a decrease in the thiol groups. Previous study confirmed the presence of the oxidative stress in COPD [10]. It was also proven that cigarette smoke contains stable compounds that undergo redox-cycle and form reactive oxygen species such as superoxide radicals, hydrogen peroxide, and hydroxyl radicals [35].

With regard to beneficial effect of *Z. multiflora* on the bronchial inflammation which was reported in traditional medicine [36], in the present study, therapeutic potentials of *Z. multiflora* and its constituent carvacrol in animals exposed to cigarette smoke (an animal model of COPD) were studied. Total WBC, eosinophils and neutrophils counts as well as IL-8 level in BALF were significantly decreased and thiol group was significantly increased in COPD groups treated with at least two higher concentrations of the extract of *Z. multiflora* and carvacrol. These findings suggest a preventive therapeutic property for *Z. multiflora* and its constituent carvacrol in the development of COPD. Previous studies have also shown anti-inflammatory effects of *Z. multiflora* and its constituents, which support the results of the present study [37,38]. Increased thiol group in BALF of COPD animals treated with two higher concentrations of *Z. multiflora* and carvacrol seen in the present study was supported by the antioxidant effect of *Z. multiflora* [16] and carvacrol [39,40] in previous studies.

The concentration-dependent effects of *Z. multiflora* and carvacrol could be another indicator of the preventive effect of the plant and its constituent, carvacrol on COPD. However, the effect of the extract and carvacrol on IL-8 was not fully concentration-dependent and there was no significant difference between the effects of three concentrations. The most probable explanation for these findings is the achievement of maximum concentration at lower concentration of the extract and carvacrol. However, the exact explanation for this observation should be examined in future studies. Carvacrol concentrations used in the present study were about 15% of *Z. multiflora* concentrations, which are lower than the actual concentration in the plant [41]. Therefore, the similar effects of carvacrol with *Z. multiflora* on lung inflammatory changes and oxidative stress suggest that carvacrol is responsible for the observed results for *Z. multiflora*.

In the present study, dexamethasone was used as positive control because it is effective in treatment of COPD by its anti-inflammatory property [42-44]. Total WBC, eosinophil and neutrophil counts, as well as IL-8 were also significantly reduced and thiol group was increased in COPD animals treated with dexamethasone. Although the effect of lower concentration of the extract and carvacrol was smaller than the dexamethasone, the preventive effects of their medium and high concentrations were similar to dexamethasone. Similar effects of dexamethasone on measured variables with the effects of *Z. multiflora* and its constituent carvacrol also support the preventive effect of the plant and carvacrol on development of COPD with anti-inflammatory mechanism. In fact, in our previous studies, the anti-inflammatory effect of *Z. multiflora* and its constituent carvacrol on sensitized guinea pigs [45,46] and the effects of the plant on systemic inflammation of cigarette smoke exposed animals [23] were shown which further support the findings of the present study.

Various pharmacological effect of *Z. multiflora* and its constituent, carvacrol on animal model of asthma, animal model of COPD, the relaxant effect of the plant and carvacrol, and its possible mechanisms were examined. In line with our previous studies, the preventive effect of *Z. multiflora* and carvacrol on lung inflammation in animal model of COPD (exposed guinea pig to cigarette smoke) was examined in the present study which is novel and showed promising results.

The results of the present study showed a preventive effect of *Z. multiflora* and its constituent carvacrol on COPD with anti-inflammatory and antioxidant mechanisms. In fact, anti-inflammatory effect of this plant was also addressed in a recent review [47]. Therefore, these results suggested an anti-inflammatory and antioxidant or preventive effect for *Z. multiflora* on animal model of COPD (exposed animals to cigarette smoke). The relaxant effect of carvacrol showed in previous studies [28,29] and the results of the present study may suggest the therapeutic potentials of the plant on COPD by both bronchdilatory and anti-inflammatory mechanisms. However, the effect of different constituents of the plant on animal model of COPD as well as the effect of the extract and its constituents on COPD patients should be examined in further studies.

## Conclusions

In conclusion, the results of the present study indicated a preventive effect of *Z. multiflora* and carvacrol on BALF total and differential WBC, BALF levels of IL-8, and thiol group of animal model of COPD which is comparable to the effect of dexamethasone at used concentrations. Therefore, the results suggest a preventive therapeutic potential for *Z. multiflora* and its constituent, carvacrol on lung inflammation and oxidative stress in COPD.
